# A bis-sulphamoylated estradiol derivative induces ROS-dependent cell cycle abnormalities and subsequent apoptosis

**DOI:** 10.1371/journal.pone.0176006

**Published:** 2017-04-14

**Authors:** Michelle Helen Visagie, Iman van den Bout, Anna Margaretha Joubert

**Affiliations:** Department of Physiology, Faculty of Health Sciences, University of Pretoria, Pretoria, Gauteng, South Africa; Columbia University, UNITED STATES

## Abstract

Clinical trials have revealed that the potential anticancer agent, 2-methoxyestradiol (2ME2) has limitations due to its low bioavailability. Subsequently, 2ME2 derivatives including (8*R*,13*S*,14*S*,17*S*)-2-ethyl-13-methyl-7,8,9,11,12,13,14,15,16,17-decahydro-6H-cyclopenta[a]phenanthrane-3,17-diyl bis(sulphamate) (EMBS) have shown improved efficacies in inducing apoptosis. However, no conclusive data exist to explain the mode of action exerted by these drugs. This study investigated the mode of action used by EMBS as a representative of the sulphamoylated 2ME2 derivatives. Hydrogen peroxide and superoxide production was quantified using dichlorofluorescein diacetate and hydroethidine. Cell proliferation and mitochondrial metabolism were investigated using crystal violet and Alamar Blue. Apoptosis was assessed using Annexin V-FITC while mitochondrial integrity was assessed using Mitocapture. Autophagy was visualised using LC3B II antibodies. The effects of EMBS on H2A phosphorylation and nuclei were visualised using phospho H2A antibody and 4',6-diamidino-2-phenylindole, dihydrochloride. Data showed that EMBS exposure leads to increased reactive oxygen species (ROS) production which is correlated with loss of cell proliferation, mitochondrial membrane damage, decreased metabolic activity, G_2_/M arrest, endoreduplication, DNA double stranded breaks, micronuclei and apoptosis induction. Treatment of EMBS-exposed cells with the ROS scavenger, N-acetyl cysteine, abrogated ROS production, cell cycle arrest and apoptosis implying an essential role for ROS production in EMBS signaling. The inhibition of c-Jun N-terminal kinase (JNK) activity also inhibited EMBS-induced apoptosis suggesting that EMBS triggers apoptosis via the JNK pathway. Lastly, evaluation of LC3IIB protein levels indicated that autophagy is not activated in EMBS-exposed cells. Our data shows that EMBS targets a pathway that leads to increased ROS production as an early event that culminates in G_2_/M arrest and apoptosis by means of JNK-signaling in cancer cells. This study suggests a novel oxidative stress-dependent mode of action for sulphamoylated derivatives.

## Introduction

2-Methoxyestradiol (2ME2), an endogenous 17β-estradiol analogue, has been shown to exert antiproliferative- and antiangiogenic effects on various tumorigenic lines including sarcoma, prostate- and breast tumorigenic cell lines [1–5]. However, preclinical rodent breast cancer studies and clinical trial data revealed that 2ME2 is not effective as an anticancer therapy due to its low bioavailability and rapid metabolism [6–8]. Therefore, derivatives of 2ME2 such as 2-methoxyestradiol-bisulphamate (2-MEBM) have been designed and have shown encouraging results in preclinical MDA-MB-231 rodent cancer models where tumour growth inhibition and resistance to metabolism were observed [7]. A number of research groups including ours have designed several novel 2ME2 derivatives, most of which include a sulphamoyl moiety. The sulphamoyl moiety reversibly interacts with carbonic anhydrase II allowing the sulphamoylated compound to bypass first pass liver metabolism and in so doing increases bioavailability by reducing its metabolism [9–11].

These compounds are highly effective in inducing cell death in a number of cancer cell lines [9,12–14]. In previous reports we have demonstrated that the 2ME2 derivative (8*R*,13*S*,14*S*,17*S*)-2-ethyl-13-methyl-7,8,9,11,12,13,14,15,16,17-decahydro-6H-cyclopenta[a]phenanthrane-3,17-diyl bis(sulphamate) (EMBS) possesses antiproliferative effects in a variety of tumorigenic cell lines including breast tumorigenic lines including the MCF-7, MDA-MD-435 and MDA-MD-231, ovarian adenocarcinoma cell line OVCAR-3, renal carcinoma cell line SN12-C and prostate carcinoma cell line DU-145 [12–14]. Furthermore, similar to 2ME2, EMBS exposure blocked cell cycle progression in the G_2_/M phase. We showed that EMBS exposure led to mitochondrial membrane depolarisation, caspase 6 and caspase 7 activation, while the extrinsic apoptotic pathway was also activated through caspase 8 [14]. Furthermore, 2ME2 exposure also resulted in increased reactive oxygen species (ROS) generation including hydrogen peroxide and superoxide [2,15]. 2ME2-dependent ROS induction was also responsible for endoreduplication in nasopharyngeal carcinoma cells via c-Jun N-terminal kinase (JNK) and mitogen-activated protein kinase family (MAPK) activation [15].

Controversy still exists around the mechanism through which 2ME2 increases ROS production. 2ME2 exposure in Ewing sarcoma cells resulted in reduced mitochondrial membrane potential, increased ROS production and activated the JNK pathway. Moreover, pre-treatment with JNK inhibitors or antioxidants largely reduced the effects of 2ME2 on cell proliferation suggesting that both ROS and the JNK pathways are involved in the mechanism of action exerted by 2ME2 [16]. However, in a separate report, 2ME2 exposure in rat sarcoma cells resulted in decreased mitochondrial membrane potential and an increase in ROS. Co-treatment with various antioxidants did not reduce the effect of 2ME2 on cell survival. Moreover, inhibition of caspases did not diminish the effect of 2ME2 further suggesting that, in these cells, 2ME2 induces apoptosis independently of caspases and ROS production, but still through a mitochondrial pathway [2]. ROS, and specifically superoxide metabolism, is regulated by the superoxide dismutase (SOD) family. Researchers have reported that ROS is induced after exposure to 2ME2 as a result of the inhibition of SOD activity [17]. However, Kachadourian., *et al* (2001) demonstrated using different assays to determine SOD activity that 2ME2 does not inhibit SOD activity [18,19]. Furthermore, they speculate that 2ME2 itself could act as a free radical after conversion to a semiquinone radical and through reaction with oxygen could form superoxide [18].

One of the major effects of oxidative stress is the formation of DNA adducts leading to DNA damage including the formation of double stranded DNA breaks (DSB) [20]. Double stranded breaks are restored through a programme of non-homologous end joining [20]. One of the proteins involved in this process is the histone H2A which is phosphorylated at the sites of DSB to act as a beacon for the assembly of the restorative protein complex [21]. This feature is used in cellular assays to assess the formation of DSB by quantifying H2A phosphorylation.

Various sulphamoylated 2ME2 derivatives have been synthesised in order to improve on the anticancer potential of 2ME2. However, except for the effects that have been measured after exposure to these compounds, conclusive data do not exist that provide a mechanism for their action within the cell. In this report the effect of EMBS exposure on ROS production was analysed. In this report we show that ROS increase is an early event after EMBS exposure. Moreover, by using antioxidants it is shown that ROS production is essential for the major effects of EMBS exposure including cell cycle arrest, mitochondrial membrane potential effects and apoptosis induction. This indicates that ROS is involved in upstream events required for JNK activation and cell cycle arrest. Furthermore, initial ROS generation is needed for subsequent loss of mitochondrial membrane potential and the secondary increase in ROS production.

## Aim and design of the study

This study investigated the mode of action utilized by EMBS as a representative of the sulphamoylated 2ME2 derivatives. Moreover, the role of ROS in the induction of ROS was demonstrated to induce cell death via apoptosis.

This research project is regarded as a preclinical *in vitro* study and data cannot directly be extrapolated to an *in vivo* environment.

## Materials and methods

### Cell lines

The estrogen receptor-positive MCF-7 cell line [22] and the estrogen receptor-negative metastatic MDA-MB-231 cell line [23] were obtained from Cellonex. (Johannesburg, South Africa). Both cell lines were propagated and maintained in a humidified atmosphere at 37**°**C and 5% CO_2_ in Dulbecco’s Minimum Essential Medium Eagle (DMEM) supplemented with 10% heat-inactivated fetal calf serum (FCS), 100 U/ml penicillin G, 100 μg/ml streptomycin and fungizone (250 μg/l). Non-tumorigenic spontaneously immortalized adherent human breast epithelial MCF-12A cells [24] were a gift from Professor Parker (Department of Medical Biochemistry, University of Cape Town, Western Cape, South Africa). MCF-12A cells were cultured in growth medium consisting of a 1:1 mixture of DMEM and Ham’s-F12 medium supplemented with 20 ng/ml epidermal growth factor, 100 ng/ml cholera toxin, 10 μg/ml insulin and 500 ng/ml hydrocortisone, 10% heat-inactivated FCS, 100 U/ml penicillin G, 100 μg/ml streptomycin and fungizone (250 μg/l).

### Reagents

All required reagents of cell culture analytical grade were purchased from Sigma (St. Louis, United States of America) unless otherwise specified. Heat-inactivated FCS, sterile cell culture flasks and plates were purchased from Sterilab Services (Kempton Park, Johannesburg, Gauteng, South Africa). Penicillin, streptomycin and fungizone were obtained from Highveld Biological (Pty) Ltd. (Johannesburg, Gauteng, South Africa). The lactate dehydrogenase assay kit, annexin V fluorescein isothiocyanate (FITC) kit and Mitocapture mitochondrial apoptosis detection kit were purchased from BIOCOM biotech (Pty) Ltd. (Clubview, Gauteng, South Africa). Hydroethidine, 2,7-dichlorofluorescein diacetate (DCFDA), thymidine, JNK inhibitor (SP600125), N-acetyl cysteine (NAC), triton X-100, paraformaldehyde and fluoromount aqueous mounting fluid were acquired from Sigma (St. Louis, United States of America). Monoclonal mouse anti-human phospho H2A antibody and LC3B II monoclonal antibody were obtained from Abcam (Cambridge, United Kingdom) and the secondary goat anti-mouse horseradish peroxidase antibody was supplied by KPL (Maryland, United States of America).

EMBS is commercially unavailable and was synthesized by iThemba Pharmaceuticals (Pty) Ltd. (Modderfontein, Gauteng, South Africa). A stock solution of EMBS dissolved in dimethyl sulphoxide (DMSO) was prepared with a concentration of 10 mM and was stored at 4°C. Previous studies conducted in our laboratory demonstrated optimal antiproliferative activity after exposure to 0.4 μM EMBS for 24 h [15]. For this reason, for all subsequent experiments, cell lines were exposed to 0.4 μM EMBS.

### Methods

#### Reactive oxygen species (ROS) generation

Hydrogen peroxide generation was assessed by measuring the fluorescence of the probe DCFDA, while superoxide generation was assessed by hydroethidine. DCFDA is a non-fluorescent probe, which, upon oxidation by hydrogen peroxide, hydroxyl radical and peroxides is converted to the highly fluorescent derivative, DCF. DFCDA does not respond to nitric oxide, superoxide or hypoclorous acid. Thus, DCFDA does not directly quantify hydrogen peroxide, but an increase in DCF fluorescence suggests an increase in hydrogen peroxide generation [25]. Superoxide oxidizes hydroethidine to form fluorescent 2-hydroethidine cation, but not ethidium [26]. Exponentially growing MCF-7, MDA-MB-231and MCF-12A cells were seeded at 500 000 cells per 25 cm^2^ flask. After a 24 h attachment period, medium was discarded and cells were exposed to medium containing 0.4 μM EMBS for different times. At termination cells were trypsinized and 1x10^6^ cells were resuspended in 1 ml PBS. Cells were incubated with 20 μM DCFDA for 25 minutes or 10 μM hydroethidine for 15 minutes at 37°C. DCF and the 2-hydroethidine cation fluorescent product fluorescence was measured with a FC500 System flow cytometer (Beckman Coulter South Africa (Pty) Ltd). Information generated from at least 10 000 cells were analyzed by means of Cyflogic version 1.2.1 software (Pertu Therho, Turko, Finland).

#### Cell number determination

The time-dependent influence of EMBS exposure on proliferation was investigated by means of crystal violet staining as described previously [13]. Crystal violet allows for the quantification of the cell number in monolayer cultures as a function of the absorbance of the dye taken up by the cells [27]. MDA-MB-231 cells were seeded in 96-well tissue culture plates (5000 cells/well) and were incubated for 24 h to allow for attachment. Medium was discarded and cells were incubated in medium containing 0.4 μM EMBS for different timepoints. Cells were fixed with 1% gluteraldehyde (100 μl) for 15 minutes at room temperature. Gluteraldehyde was discarded and cells were stained using 100 μl 0.1% crystal violet at room temperature for 30 minutes. Excess crystal violet was discarded and the 96-well plate was washed by rinsing the plate under running water for 2 minutes. Triton X-100 (0.2%; 200μl) was added and incubated at room temperature for 30 minutes. Solubilised crystal violet solution (100 μl) was transferred to a new microtitre plate. The absorbance was determined at 570 nm using an EL_x_800 Universal Microplate.

#### Mitochondrial membrane potential

Mitochondrial membrane potential was measured using Mitocapture technology [12]. The mitochondrial membrane potential is an indicator of mitochondrial function. Reduced mitochondrial membrane potential has been implicated in intrinsic apoptosis induction that is dependent on ROS induction [28]. MDA-MD-231 cells (500 000) were seeded in 25 cm^2^ flasks. After 24 h, cells were exposed to 0.4 μM EMBS for different times. Subsequently, cells were detached using trypsin and centrifuged at 500 *g*. Cells were resuspended in 1 ml of diluted Mitocapture solution (1 μl mitocapture: 1 ml pre-warmed incubation buffer) and incubated in a humidified atmosphere (37°C, 5% CO_2_). After 20 minutes, cells were centrifuged at 500 x *g*, the supernatant discarded and cells resuspended in 1 ml of pre-warmed incubation buffer (37°C). Cells were analyzed with a FC500 System flow cytometer. Data from at least 10 000 cells were analyzed by means of Cyflogic version 1.2.1 software.

#### Apoptosis induction

Apoptosis induction was analysed using flow cytometry and annexin V-FITC. MDA-MB-231 cells (500 000) were seeded in 25 cm^2^ flasks. After exposure to 0.4 μM EMBS, cells were trypsinised and cells were resuspended in 1 ml of 1x binding buffer and centrifuged at 300 x *g* for 10 minutes. Supernatant was removed and cells were resuspended in 100 μl of 1x binding buffer. Annexin V-FITC (10 μl) was added and incubated for 15 minutes in the dark at room temperature. After 15 minutes, cells were washed by adding 1 ml of 1x binding buffer and centrifuged at 300 x *g* for 10 minutes. Supernatant was carefully removed and cells were resuspended in 500 μl of 1x binding buffer solution. Immediately prior to analysis, 12.5 μl of propidium iodide (PI) (40 μg/ml) was added and samples were mixed gently and analysed using the FC500 System flow cytometer. Data from at least 10 000 cells were analyzed with CXP software. Distributions of cells within the quadrants were calculated with Cyflogic version 1.2.1 software.

#### Mitochondrial metabolic activity

The influence of EMBS on metabolic activity was demonstrated over time using Alamar Blue (also known as resazurin) and fluorometry [29]. Alamar blue is stable and non-toxic and thus enables the continuous quantification of metabolic activity over time. Alamar blue (non-fluorescent and blue) is converted by mitochondrial enzymes to the reduced form, resorufin (highly fluorescent and red) [30]. MDA-MB-231 cells were seeded at 5 000 cells/well into 96 well plates. After 24 h, cells were exposed to 5% Alamar Blue and 0.4 μM EMBS. Samples were incubated at 37°C and fluorescence was measured at an excitation wavelength of 570 nm and reference wavelength 600 nm at different timepoints.

#### Cell cycle progression

Flow cytometry was utilized to investigate the deoxyribonucleic acid (DNA) content to determine the effects of EMBS on cell cycle distribution and confirmation of apoptosis induction [31]. MDA-MB-231 cells (500 000) were seeded in a 25 cm^2^ flask. Medium was discarded and cells were incubated in medium containing 0.4 μM EMBS for different timepoints. Subsequently, cells were trypsinized and 10^6^ cells were centrifuged for 5 minutes at 300 x *g*. The pellet was resuspended twice in ice-cold phosphate buffer solution (PBS). The supernatant was discarded and cells were resuspended in 200 μl of ice-cold PBS containing 0.1% FCS. Ice-cold 70% ethanol (4 ml) was added in a drop-wise manner and cells were stored at 4°C for 24 h. Cells were pelleted by centrifugation for 5 minutes. The supernatant was removed and cells were resuspended in 1 ml of PBS containing PI (40 μg/ml), 0.1% triton X-100 and RNase A (100 μg/ml) and incubated at 37°C, 5% CO_2_ for 45 minutes. Cells were analysed by means of FC500 System flow cytometer. Data from at least 10 000 cells were captured with CXP software and analyzed with Cyflogic software.

#### H2A phosphorylation

Oxidative stress frequently results in DNA damage associated with phosphorylated H2A. H2A is phosphorylated, activated and recruited to sites of double stranded breaks (DSB) after oxidative damage as part of the DNA repair mechanism [32]. For confocal imaging of phosphorylated H2A, cells (50 000) were plated on sterile glass coverslips in 24 well plates. The next day, cells were exposed to 0.4 μM EMBS. Cells were exposed for 4 h and 24 h respectively and were subsequently fixed in 2% paraformaldehyde for 15 minutes. Slides were washed with PBS and permeabilized with 0.2% triton X-100. Coverslips were subsequently blocked with 2% BSA in PBS for 1 h, incubated with primary antibody against phosphorylated H2A for 1 h at room temperature, washed with PBS, and incubated with FITC-conjugated anti-mouse secondary antibodies and 4',6-diamidino-2-phenylindole, dihydrochloride (DAPI) for 1 h at room temperature. After washing with PBS, coverslips were mounted in mounting fluid. Slides were examined using a Zeiss LSM510 Meta confocal microscope furnished with a 63x magnification oil objective. Representative images were made of each condition and the experiment was repeated three times. Images were analysed using Image J software where after global adjustment H2A phosphorylation was quantified using rawIntDev values divided by number of cells per image. At least 100 cells were measured per condition in each experiment.

#### Autophagy induction

During autophagy organelles and proteins are sequestered into autophagic vesicles that are subsequently degraded through fusion with lysosomes and recycled for sustainable cell maintenance [33]. In order to measure the induction of autophagy microtubule-associated protein B-light chain 3 II (LC3BII) levels were determined by western blot analysis. MDA-MB-231 cells (200 000) were plated in 6 well plates, grown overnight and exposed to EMBS for 4 h, 10 h, 14 h and 24 h. As a positive control for LC3B II accumulation cells were treated with 100 nM bafilomycin A overnight. At termination, medium was removed and cells were carefully washed once in PBS. Lithium dodecyl sulphate (LDS) loading buffer with 2.5% β-mercaptoethanol was heated to 80°C and 150 μl of sample was plated into each well. Cells were scraped together and passed through a syringe to help lyse cells. Cell lysates were heated to 80°C for 10 minutes and then centrifuged at 11000 rpm for 15 minutes. Supernatants were loaded onto 4–12% SDS-PAGE gels. After resolving proteins on gel, they were blotted onto a 0.2 μm polyvinylidene fluoride (PVDF) membrane overnight at 60 V. Membranes were blocked in 2% blocking reagent before being incubated with 1:1000 dilution of monoclonal LC3B antibody or 1:5000 dilution of the monoclonal actin antibody for 1 h and secondary goat anti-mouse horseradish peroxidase antibody for 1 h with washes in between with PBS-Tween. Proteins were visualised using enhanced chemiluminescence substrate and the Biorad Chemidoc MP system (Bio-Rad Laboratories, Inc. California, United States of America). Bands were quantified using Imagelab software and ratios of LC3B II to actin were calculated using Image Lab Software developed by Bio-Rad Laboratories, Inc.

### Statistics

For all experiments data were obtained from 3 independent experiments. Graphs represent the mean of three experiments with error bars depicting the standard error of the mean. Each independent experiment for crystal violet staining and extracellular lactate dehydrogenase quantification had a technical repeat of 6. Each independent Alamar Blue experiment had 3 technical repeats. Data were statistically analyzed for significance using a two-tailed Student’s *t*-test. *P*-values smaller than 0.05 were regarded as statistically significant and were indicated by an asterisk (*). Fluorescent measurement was expressed as a ratio of the value measured for the EMBS-treated cells compared to vehicle-treated exposed cells (mean relative fluorescence). Flow cytometry analysis involved data from at least 10 000 events that were repeated thrice. Bands produced in western blot were quantified using Imagelab developed by Bio-Rad Laboratories, Inc. (California, United States of America) and ratios of LC3B II to actin were calculated.

## Results

### EMBS induces oxidative stress in breast cancer cell lines

**Fig 1:**
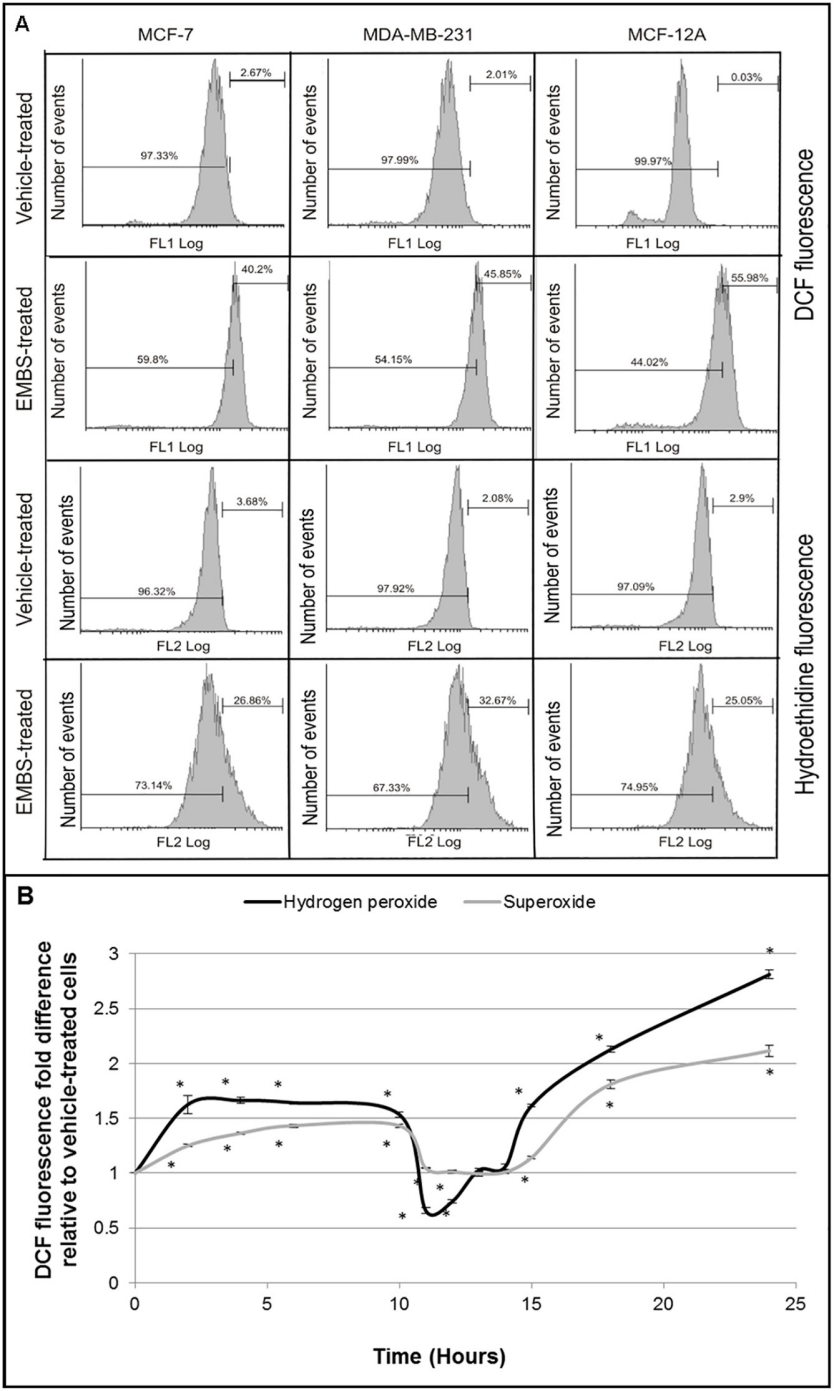
EMBS exposure results in oxidative stress. (A) Flow cytometry of 2,7-dichlorofluorescein diacetate- and hydroethidine-stained cells to determine the influence of EMBS (0.4 μM, 24 h) exposure on hydrogen peroxide and superoxide production in MCF-7-, MDA-MB-231- and MCF-12A cells. Histograms were gated for positive- and negative staining and the percentage positive cells are included in each graph. (B) MDA-MB-231 cells were exposed to 0.4 μM EMBS for the indicated timepoints. Hydrogen peroxide (dark grey line) and superoxide (light grey line) were measured. The graph represents the average of 3 independent experiments with error bars representing s.e.m. Values represent the fold-change compared to cells at 0 h. An asterisk (*) demonstrates a statistically significant *P* value <0.05 when compared to vehicle-treated cells.

The influence of EMBS (0.4 μM) on ROS was initially investigated by determining the intracellular levels of hydrogen peroxide and superoxide 24 h after exposure. At this time both, hydrogen peroxide and superoxide generation increased in MCF-7 cells (40% and 27%), MDA-MB-231 cells (46% and 33%) and in the non-tumorigenic MCF12A cell line (56% and 25%) (Fig 1A).

To identify the sequence of events resulting in EMBS-induced apoptosis, ROS production was measured over time. MDA-MD-231 cells were exposed to 0.4 μM EMBS and hydrogen peroxide and superoxide production were measured at several timepoints up to 24 h after exposure (Fig 1B). While the absolute levels of hydrogen peroxide and superoxide varied, a similar trend was observed over time for both ROS species. Both increased significantly over the first 4 h with hydrogen peroxide levels increasing rapidly up to 1.7 times above the control and then plateauing, while superoxide levels increased more gradually over the same time up to 1.3 times above initial levels. Hydrogen peroxide levels decreased suddenly and dramatically at 10 h (down to 0.5 compared to initial levels) only to recover from 12 h onwards. These levels subsequently increased consistently over the remaining time up to nearly 3-fold over initial measurements. Superoxide levels also decreased after 10 h, but more gradually and less pronounced with a recovery followed by a plateau at 1.7 fold over initial measurements after 16 h.

### EMBS treated cells undergo apoptosis correlated with decreased mitochondrial membrane potential

**Fig 2:**
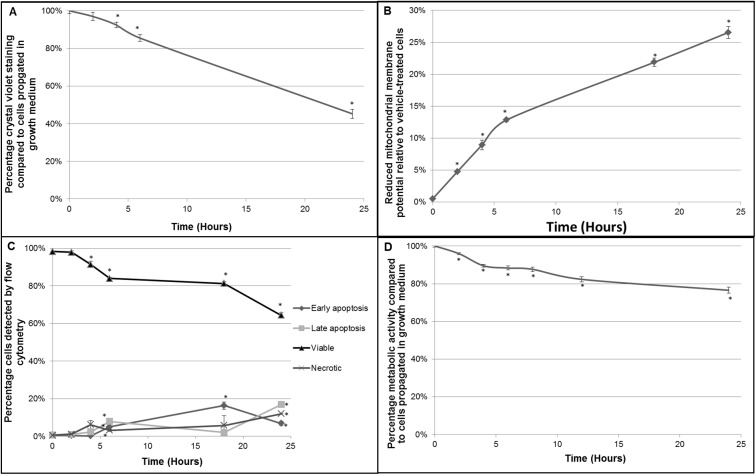
EMBS-treated cells undergo apoptosis correlated with decreased cell viability, metabolic activity and mitochondrial membrane potential. (A) MDA-MB-231 cells were exposed to 0.4 μM EMBS for the indicated timepoints and stained with crystal violet. The graph represents the average of three independent experiments with error bars representing s.e.m. Values represent the absorbance measurements at 570 nm. An * demonstrates a statistically significant *P* value <0.05 when compared to vehicle-treated cells. (B) MDA-MB-231 cells were exposed to 0.4 μM EMBS for the indicated timepoints. Subsequently, Mitotracker was quantified using flow cytometry. The graph represents three independent experiments with error bars of s.e.m. Apoptosis was measured relative to vehicle-treated cells. An * demonstrates a statistically significant *P* value <0.05 when compared to vehicle-treated cells. (C) Annexin V/PI staining was used to determine the number of viable (▲), necrotic (X), early- (♦) and late apoptotic cells (■) at different timepoints after EMBS exposure. The graph represents the average of three independent experiments with error bars representing s.e.m. An * demonstrates a statistically significant *P* value <0.05 when compared to vehicle-treated cells. (D) To measure metabolic activity cells were exposed to EMBS and simultaneously incubated with Alamar blue. At indicated timepoints Alamar Blue was measured and the percentage reduction in exposed cells was calculated relative to that in control cells. The graph represents the average of three independent experiments with error bars representing s.e.m. An * demonstrates a statistically significant *P* value <0.05 when compared to vehicle-treated cells.

To determine whether oxidative stress is a precursor for EMBS-induced apoptosis via mitochondrial membrane potential loss, both mitochondrial membrane potential and apoptosis levels were measured over time. Firstly, cell survival was measured using crystal violet in cells exposed to 0.4 μM EMBS (Fig 2A). Data showed that there was a gradual decrease in proliferation starting at 2 h after exposure that continued to 6 h and eventually to 24 h with a loss in cell number compared to cells propagated in growth medium to approximately 50%. Mitochondrial membrane potential depolarisation statistically significantly increased by about 10% over the first 6 h after exposure to EMBS. A gradual incline (20%) in reduced mitochondrial membrane depolarisation was subsequently measured until 24 h (Fig 2B). Apoptosis was measured using PI/Annexin V (Fig 2C). Early and late apoptosis sharply increased after 4 h after which early apoptosis increased further, while late apoptosis remained stable. The population of cells present in early apoptosis dropped 18 h after exposure and late apoptosis increased indicating cells were committed to the apoptotic pathway. Data revealed minimal induction of necrosis. Alamar blue was used to determine if EMBS influences the metabolic activity of the cell (Fig 2D). Alamar Blue reduction decreased steadily from 2 h onwards after EMBS exposure ending at a 30% decrease in fluorescence after 24 h. Therefore, EMBS exposure resulted in increased ROS production that is tightly correlated with decreased mitochondrial membrane potential and electron transport efficiency along with increased apoptosis.

### Oxidative stress is essential for EMBS induced cell death

**Fig 3:**
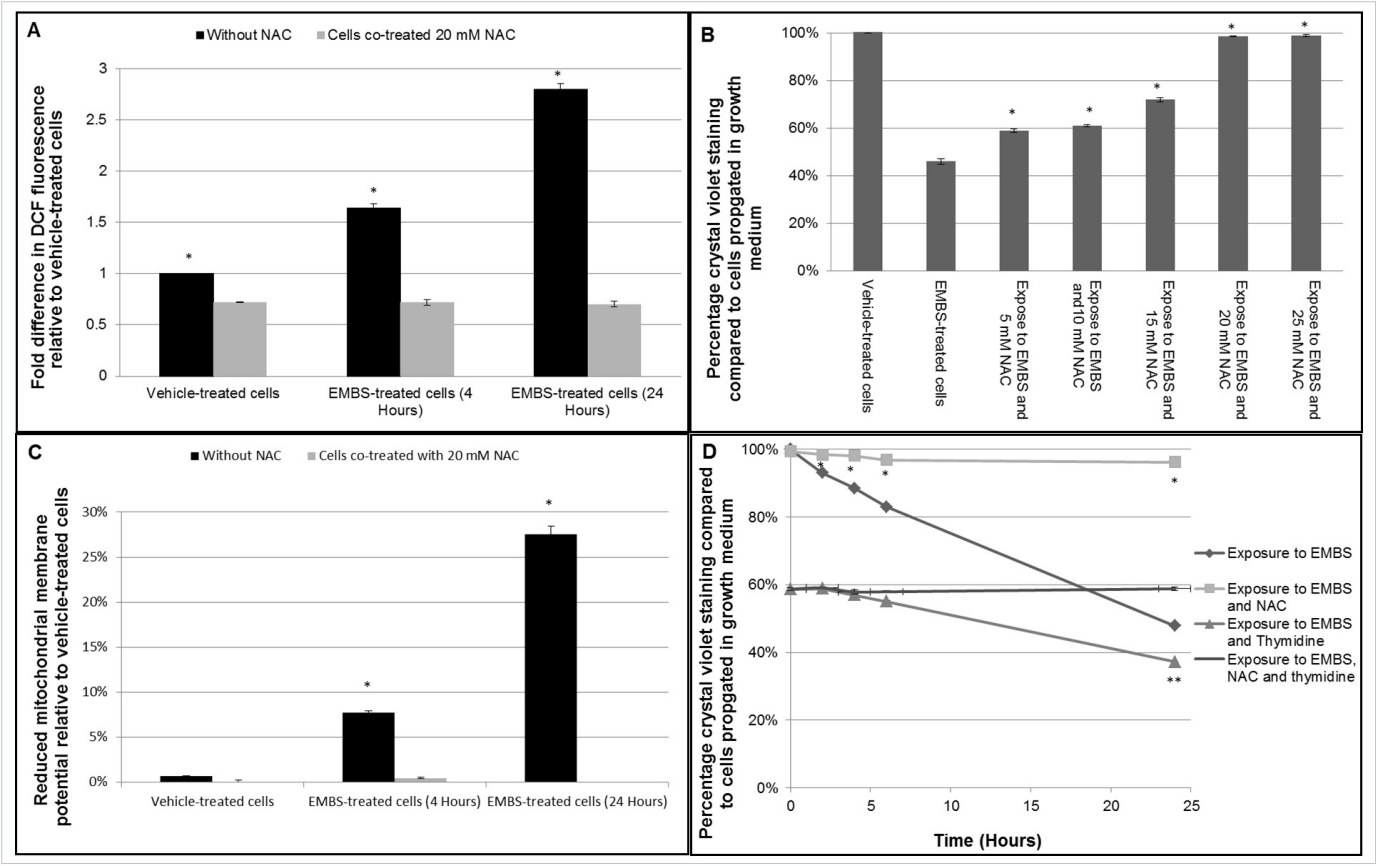
Antiproliferative and apoptotic effects exerted by EMBS are ROS-dependent. (A) Hydrogen peroxide production of EMBS-treated cells in the presence of absence of 20 mM NAC. The graph represents the average of 3 independent experiments with error bars representing s.e.m. Values represent fold-change compared to vehicle-treated cells. A * demonstrates a statistically significant *P* value <0.05 when compared to EMBS-treated cells. (B) Crystal violet staining demonstrating the dose-dependent inhibition of NAC on the antiproliferative activity of 0.4 μM EMBS measured after 24 h. Cell growth is expressed as a percentage compared to cells propagated in control growth medium. The graph represents the average of 3 independent experiments with error bars representing s.e.m. An * demonstrates a statistically significant *P* value <0.05 when compared to EMBS-treated cells. (C) Mitochondrial membrane potential of EMBS-treated cells in the presence or absence of 20 mM NAC. Cells treated with EMBS alone (dark grey bar) or EMBS and NAC (light grey bar) were analysed using Mitotracker. The graph represents three independent experiments with error bars of s.e.m. An * demonstrates a statistically significant *P* value <0.05 when compared to cells exposed to 0.4 μM EMBS and 20 mM NAC. (D) MDA-MB-231 cells were either left unblocked or were blocked in G_1_/S by thymidine and were exposed EMBS alone or together with 20 mM NAC. The graph represents the average results of crystal violet of three independent experiments with error bars representing s.e.m. * demonstrates a statistically significant *P* value of <0.05 when compared to EMBS-treated cells. An ** demonstrates a statistically significant *P* value <0.05 when compared to cells treated with EMBS, thymidine and NAC.

As discussed above there are opposing data regarding the role of ROS in 2ME2-induced apoptosis. Therefore, in this study ROS production induced by EMBS was inhibited by co-treating cells with the antioxidant, NAC. Cleavage of NAC at the acetyl group liberates reduced cysteine resulting in sustained production of glutathione, an essential antioxidant. Glutathione neutralizes ROS by providing a sulfyhydryl group which serves as a reducing equivalent. Glutathione also functions as a substrate in order to promote the antioxidant activity of glutathione peroxidases [34]. To assess whether the antioxidant NAC inhibits EMBS-induced ROS, cells were co-treated with NAC and EMBS and hydrogen peroxide levels were measured (Fig 3A). Hydrogen peroxide levels were increased within 4 h after EMBS treatment, but co-treatment with NAC abrogated this increase. To determine whether there is a causal link between ROS production by EMBS and cell death, cells were treated with a concentration range of NAC in the presence of EMBS and the effects on proliferation were measured using crystal violet (Fig 3B). Data revealed an increase in cell viability that was correlated with increased concentrations of NAC. Furthermore, NAC abolished mitochondrial membrane potential depolarisation induced by EMBS after 4 h and 24 h (Fig 3C).

Previous studies have shown that EMBS-treated cells accumulate at the G_2_/M border in the cell cycle [12,14]. To determine if the block at the G_2_/M border is essential for EMBS induced cell death, cells were prevented from accumulating at the G_2_/M border by pretreatment with thymidine to block cells at the G_1_/S border instead. Subsequently, cells were treated with NAC in the presence of EMBS in order to inhibit ROS accumulation (Fig 3D). Crystal violet data showed that EMBS reduced cell viability by 50% in non-blocked cells. In addition, cell viability of thymidine-blocked cells was reduced by 40% after EMBS treatment. In both cycling cells and thymidine-blocked cells, 20 mM NAC treatment completely abrogated the effect of EMBS suggesting that ROS production is essential for the induction of cell death after EMBS, while the blockage of cells at the G_2_/M border is not an essential prerequisite for the induction of cell death. Moreover, data indicate that thymidine had no effect on hydrogen peroxide generation after EMBS exposure suggesting that ROS generation by EMBS treatment is upstream from the cell cycle arrest (S1 Fig).

### Oxidative stress induced by EMBS is responsible for the G_2_/M cell cycle block leading to endoreduplication

**Fig 4:**
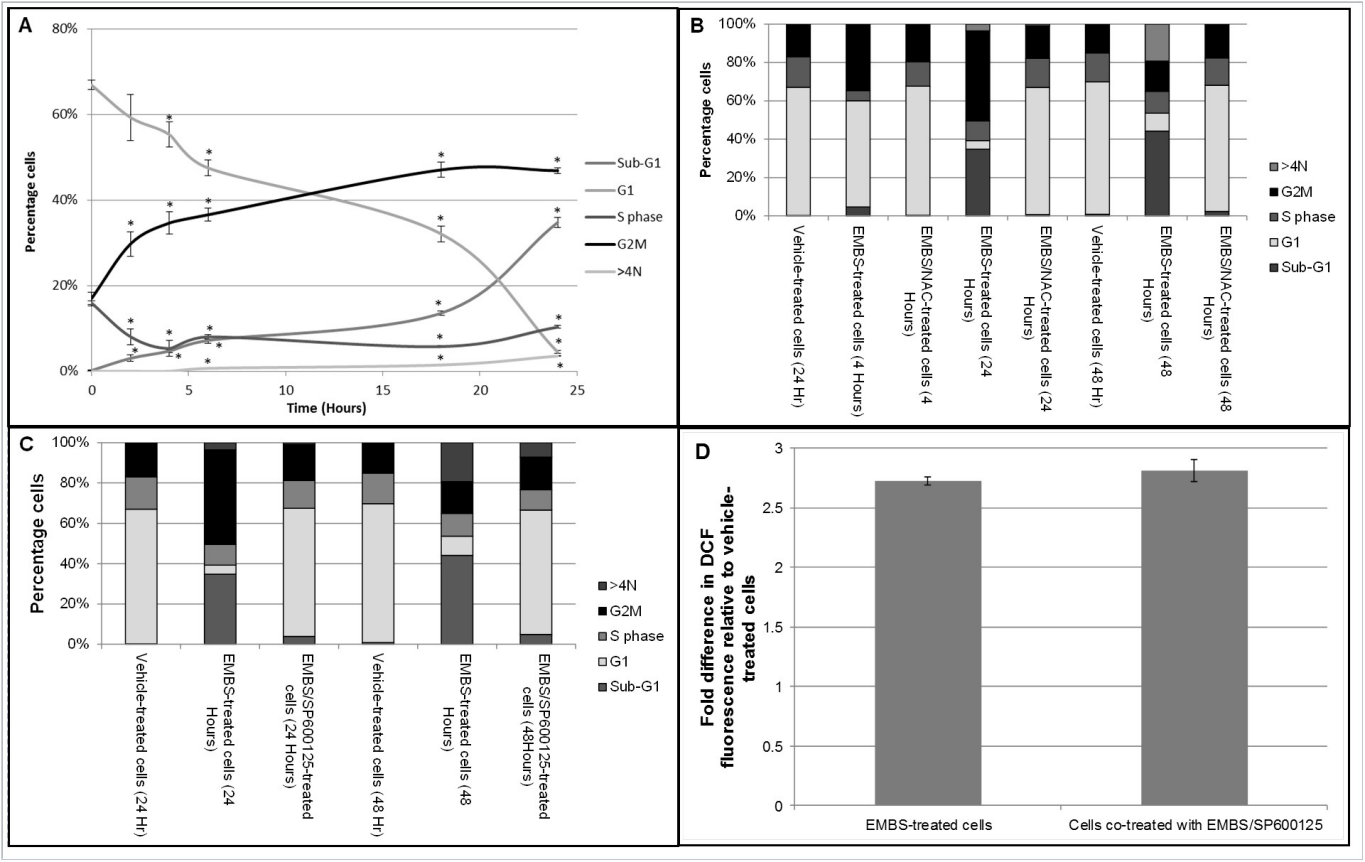
EMBS induces ROS/JNK-dependent apoptosis, G2/M block and endoreplication. (A) Cell cycle progression was analysed using PI in cells treated with EMBS alone, EMBS together with NAC or EMBS together with the JNK inhibitor, SP600125. The graph represents three independent experiments showing the percentage of cells in each of five categories. Error bars represent s.e.m. An * demonstrates a statistically significant *P* value of <0.05 when compared to vehicle-treated cells. (B) Cell cycle progression analysis of analysis of EMBS-treated MDA-MB-231 cells in the presence or absence of NAC after 4 h, 24h and 48 h. The graph represents three independent experiments showing percentage of cells in each of four categories. (C) Cell cycle progression analysis of analysis of EMBS-treated MDA-MB-231 cells in the presence or absence of Jnk inhibitor (SP600125) after 4 h, 24 h and 48 h. The graph represents three independent experiments showing percentage of cells in each of four categories. (D) Flow cytometry was conducted to investigate if JNK inhibition influences EMBS-induced ROS production. Cells were either treated with 0.4 μM EMBS for 24 h in the presence or absence of the JNK inhibitor (SP600125) after which DCF fluorescence was measured. The graph represents the average fold change between treated and vehicle-treated cells of 3 independent experiments with error bars representing s.e.m.

To determine if oxidative stress induced by EMBS is correlated to the EMBS-induced cell cycle block at the G_2_/M border, the cell cycle status of cells were determined over time (Fig 4A). Within 6 h after exposure a rapid accumulation of cells at G_2_/M was observed along with a reduction in cells in the G_1_ phase. A larger reduction of cells in the G_1_ phase was observed from 18 h onwards that correlated with an increase in cells present in the sub-G_1_ population suggesting that apoptosis was induced at this time. This data suggest that there is a close correlation between the initial increase in oxidative stress and the increase in cells at the G_2_/M border. Furthermore, cell cycle data revealed that exposure to EMBS resulted not only in the blockage of cells at the G_2_/M border, but also an increase of cells with a DNA content of more than 4N (Fig 4B). The >4N content increased even more prominently after exposure to EMBS for 48 h, while the number of cells blocked at G_2_/M were reduced when compared to cells exposed for 24 h. There was also an increase in the sub- G_1_ population. Importantly, when cells were co-treated with the antioxidant NAC, both the G_2_/M block and the presence of >4N cells were abolished. When cells were pretreated with a JNK inhibitor, the induction of a G_2_/M block was largely abolished after 24 h and 48 h although it was less effective than the addition of NAC (Fig 4C). This data suggests that EMBS induces a G_2_/M block in cells via the induction of ROS which ultimately leads to cells death or endoreduplication. To determine whether JNK activation is involved in EMBS-dependent ROS production, hydrogen peroxide levels were quantified in EMBS exposed cells co-treated with the JNK inhibitor (Fig 4D). Data demonstrated that the JNK inhibitor had no effect on hydrogen peroxide levels induced by EMBS, suggesting that ROS production occurs upstream from JNK activation after EMBS exposure.

### EMBS exposure leads to H2A phosphorylation and nuclear deformities

**Fig 5:**
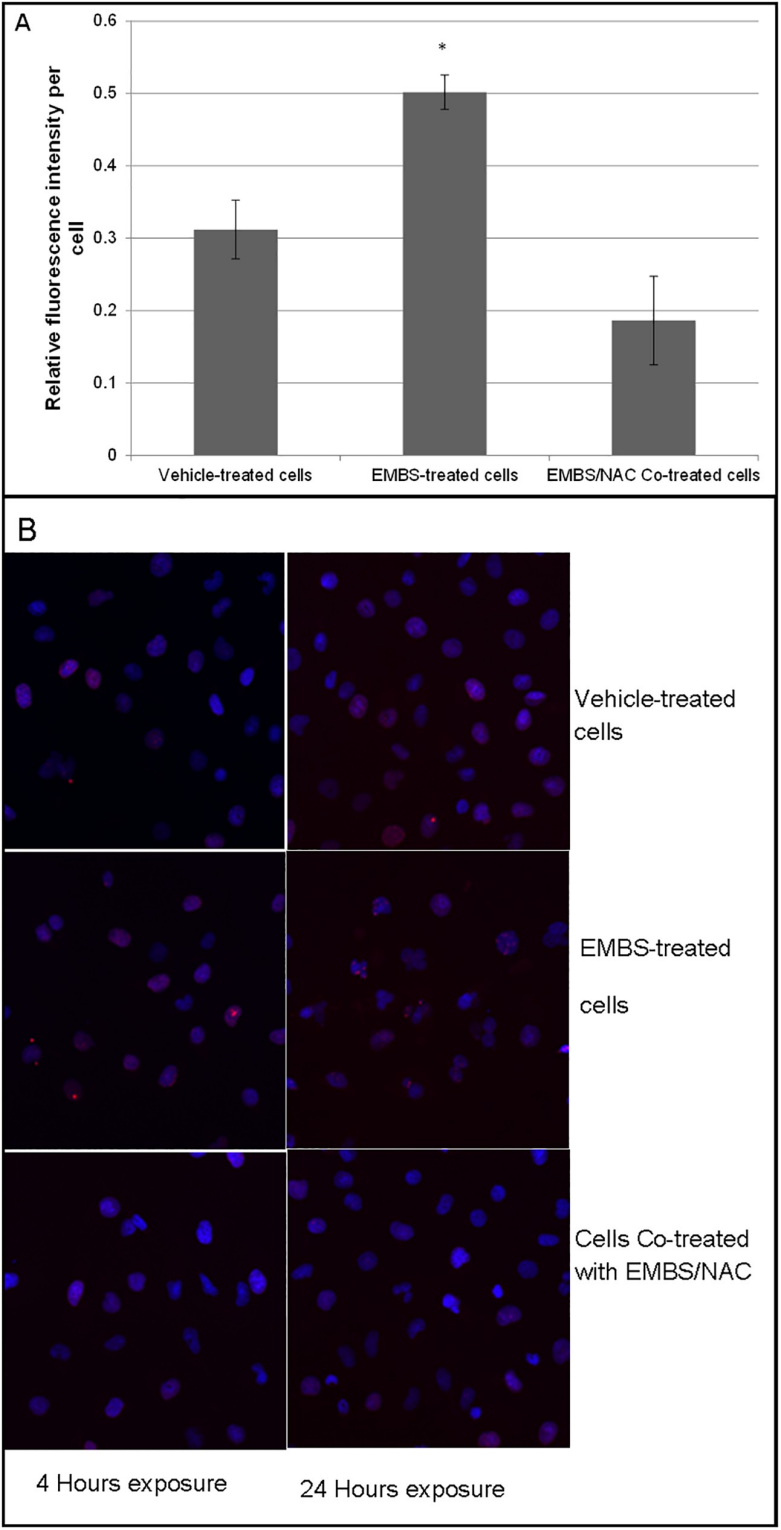
EMBS induces DNA double strand breaks and endoreduplication. (A) Confocal images of cells treated for 4 h with 0.4 μM EMBS or EMBS and 20 mM NAC were quantified by determining pixel values for H2A staining divided by the number of cells per image. The average of 100 cells per treatment was used and the average of three independent experiments are represented with s.e.m. represented by the error bars. A significant difference between the DMSO treated and EMBS treated cells was observed at a *P*-value of 0.25. (B) Cells incubated with EMBS or EMBS and NAC for 24 h were stained for phosphorylated H2A (red) and DAPI (blue). Representative images show an increase in the number of deformed nuclei in EMBS treated cells, while these were mostly absent in control or NAC-treated cells.

The blockage in cell cycle progression and the induction of endoreduplication by EMBS can be a result of genomic instability brought about by ROS induced DNA double stranded breaks. DSB attract the protein H2A which upon binding is phosphorylated [35]. Therefore, DSB can be quantified by measuring H2A phosphorylation. Cells exposed to EMBS for 4 h or 24 h were analysed for H2A phosphorylation by means of confocal microscopy (Fig 5A). Expression was normalised to cell number using DAPI and results show that after 4 h of EMBS exposure there was a small, statistically significant increase in H2A phosphorylation which was blocked by co-treatment with NAC (Fig 5B). Interestingly, after 24 h, DAPI staining of the nuclei revealed an accumulation of cells with deformed nuclei resembling multiple nuclei or micronuclei similar to that seen after radiation exposure. These nuclear deformities are also present when cells undergo endoreduplication. Again this effect was abrogated by the co-treatment of cells with 20 mM NAC. Therefore, EMBS exposure leads to a ROS-dependent increase in DNA DSB and endoreduplication.

### EMBS does not induce autophagy

**Fig 6:**
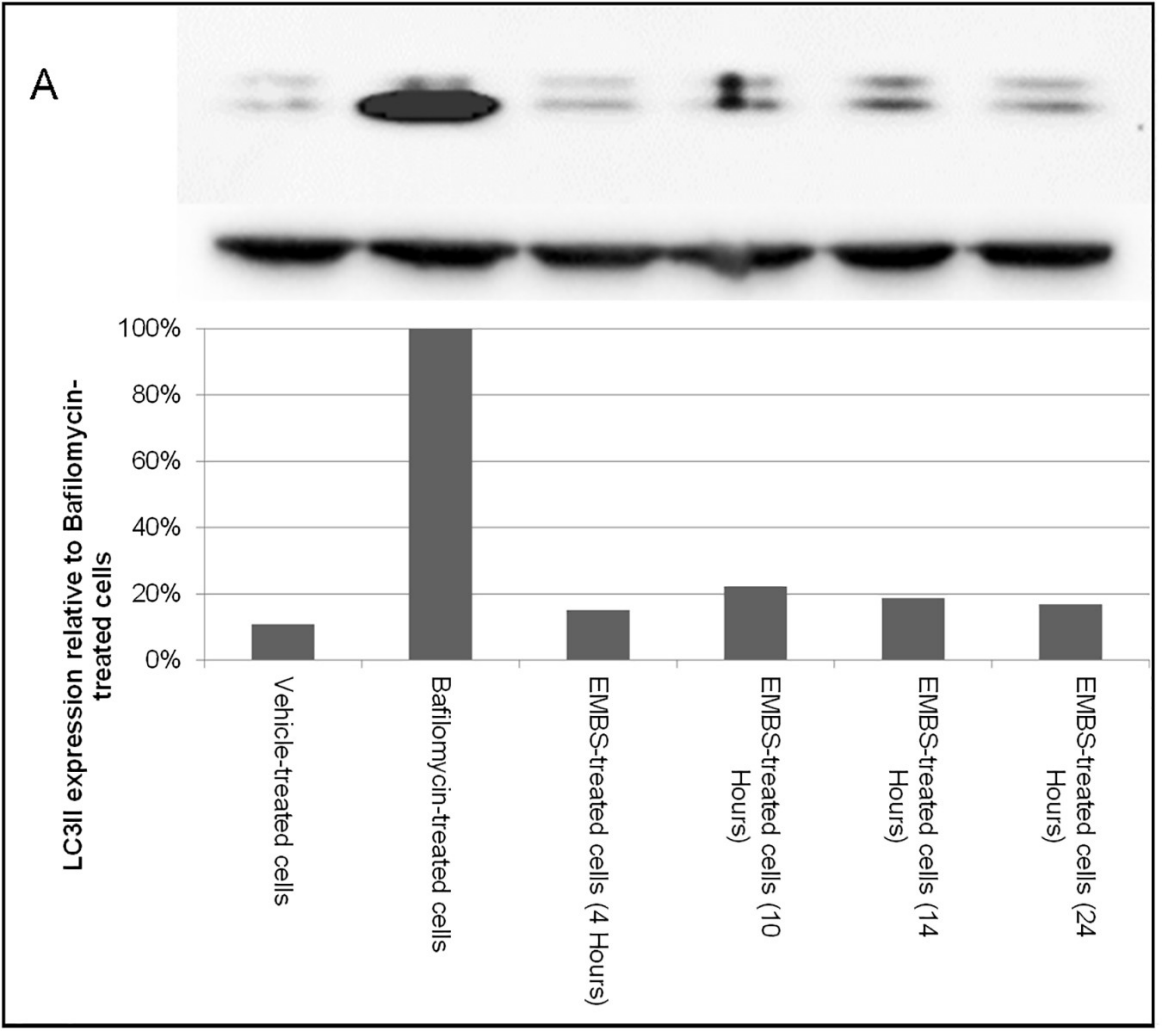
EMBS does not induce autophagy. (A) Autophagy was measured by determining the levels of LC3B II by western blot. Cells were treated with DMSO as control (lane 1), bafilomycin A1 (lane 2) as positive control or EMBS for 4 (lane 3), 10 (lane 4), 14 (lane 5) or 24 (lane 6) h. Total protein loading was measured by western blot against actin. The graph represents expression as a percentage of the positive control that represents cells exposed to bafilomycin A1. Both blot and graph are representative of three independent experiments.

While autophagy is linked to starvation-induced survival there is data suggesting that starvation may induce 5' adenosine monophosphate-activated protein kinase (AMPK)-dependent autophagy induction via ROS production [23]. To determine if EMBS-induced ROS production leads to increased autophagy, LC3B II protein levels were analysed. Cells were exposed to 0.4 μM EMBS for the time points indicated and the ratio of LC3B II to actin expression was determined (Fig 6A). Western blot data indicated that EMBS exposure did not result in increased levels of LC3B II protein. As expected, bafilomycin A exposure did lead to an increase in LC3B II levels, since it inhibits the fusion of autophagosomes with lysosomes and the flux of LC3B. Therefore, EMBS does not induce LC3B II levels suggesting that autophagy is not involved in EMBS-induced cell death.

## Discussion

Previous studies have shown that EMBS inhibits proliferation and cell cycle progression by blocking cells at the G_2_/M border [11,14]. It has been proposed that these sulphamoylated compounds function by targeting the microtubules based on *in vitro* microtubule polymerisation assays [12,14,36]. However, studies on the mechanism of action pertaining to 2ME2 have shown an essential role for ROS production in the induction of cell death [15,16]. Therefore, this study investigated the early events after EMBS exposure to determine the role of ROS production on the cellular effects exerted by the compounds. Moreover, we elucidated a pathway leading from EMBS exposure to increased ROS production resulting in several effects including mitochondrial membrane depolarisation, metabolic activity reduction, G_2_/M arrest, JNK-dependent apoptosis and endoreduplication.

To determine if EMBS induces ROS production both hydrogen peroxide- and superoxide levels were quantified. Data indicate that EMBS increases ROS production significantly after 24 h in several breast cell lines. A more detailed analysis revealed a biphasic temporal induction of ROS after EMBS exposure. The initial ROS increase was correlated with reduced mitochondrial membrane potential, reduced metabolic activity and a small decrease in cell proliferation. Loss of mitochondrial membrane potential has been proposed to be essential for an increase in ROS observed in 2ME2-exposed cells, since treatment with an inhibitor of the mitochondrial respiratory chain, rotenone, inhibited 2ME2-induced ROS and apoptosis [2,15,16]. Unexpectedly, we observed a substantial decrease in ROS production approximately 10 h after EMBS exposure. We speculate that the initial increase in ROS levels is due to a direct effect exerted by EMBS which initiates the signaling pathway resulting in the subsequent depolarisation of the mitochondrial membrane leading to the second increase in intracellular ROS generation. Furthermore, the decrease in ROS production after 10 h exposure to EMBS might represent the cells’ attempt to neutralize the ROS production through antioxidant defence activity. The later and more substantial ROS increase is correlated with a cell cycle arrest at the G_2_/M border and increased apoptosis induction. This suggests that ROS production is tightly correlated with the G_2_/M arrest and apoptosis induction.

The possibility that ROS is not only correlated to, but is causally linked to the G_2_/M arrest and apoptosis induction, was subsequently investigated. This was done by exposing cells to EMBS and NAC concurrently and determining the effects on the mitochondrial membrane potential, cell proliferation and cell cycle progression. The effects of EMBS on proliferation, mitochondrial membrane potential, cell cycle arrest and apoptosis induction were abrogated suggesting that ROS production by EMBS is essential for the induction of cell death via apoptosis and cell cycle arrest.

While this study has demonstrated that ROS production is essential for cell cycle arrest and apoptosis induction by EMBS, the mechanism through which ROS is increased remains unclear. Reports have suggested that the increase in ROS after 2ME2 exposure is due to the inhibition of the superoxide dismutase SOD2 [17]. However, others have shown that *in vitro*, SOD inhibition cannot be responsible for increased ROS generation [18,19]. Moreover, it has been suggested that 2ME2 could also act as a radical itself if it is converted in cells to a semiquinone radical through a reaction with cytochrome P450. In addition, it has been shown that certain estrogens can form DNA adducts which can lead to impaired respiratory chain function [18]. Therefore, a mechanism where EMBS itself becomes a radical cannot be excluded and further investigation will have to be conducted to determine if this does indeed occur. SOD activity also needs to be analysed to determine if EMBS induces ROS production by inhibition of SOD.

Data from this study show that ROS production is an early event which occurs simultaneously to a decrease in mitochondrial membrane potential, while significant increases in apoptosis induction were only observed after the second more substantial rise in ROS production 14 h after EMBS exposure. Interestingly, the other early event occurring after EMBS exposure is an increase in cells blocked within the G_2_/M fraction (4N) of the cell cycle. After 4 h of exposure a significant increase in blocked cells was observed increasing further up to 24 h. However, after 48 h the fraction of blocked cells decreased with a concomitant increase in cells that underwent endoreduplication (>4N). NAC treatment completely abrogated these effects suggesting that even the very early block in cell cycle is dependent on the production of ROS. Furthermore, ROS production does not depend on a G_2_/M arrest since cells blocked G1/S by thymidine still presented with increased hydrogen peroxide levels after EMBS exposure. Therefore, EMBS induces increased ROS production resulting in subsequent G_2_/M arrest.

A number of papers suggest that 2ME2 and EMBS bind to microtubules which results in a G_2_/M block. These reports are mostly based on *in vitro* tubulin polymerisation assays [1,36,37]. *In vitro* assays show that 2ME2 and EMBS prevent tubulin polymerisation although tubulin preparations including microtubule associated proteins are resistant to the effect of 2ME2 [37, 38]. In the same study cells incubated with 2ME2 for 6 h showed decreased growth rate and growth duration for microtubules while they spend more time paused resulting in an overall decrease in tubule dynamicity of 43% [39]. However, this study suggests that EMBS primarily targets ROS production leading to G_2_/M arrest and subsequent cell death induction via apoptosis.

Endoreduplication is a well-documented phenomenon where DNA replication occurs without cell division. This occurs when cells are arrested by the mitotic spindle assembly checkpoint or if cells fail to pass through cytokinesis [15]. 2ME2 was shown to induce endoreduplication which was dependent on mitochondrial oxidative stress [15]. Our data show that EMBS also induces endoreduplication in a ROS-dependent manner. As mentioned above this mostly appears in cells exposed to EMBS for 48 h where cells blocked in the G_2_/M phase were dramatically reduced. Together this data suggest that EMBS induces a blockage in the G_2_/M phase of the cell cycle which results in cells either initiating apoptosis or escape cell death through endoreduplication.

Several studies have shown that 2ME2 induces hypoxia inducible factor 1α (HIF1α) accumulation leading to the activation of the JNK pathway [16,32,40]. JNK integrates extracellular stress signals and induces apoptosis in response to these signals unlike extracellular regulated kinase (ERK) which integrates extracellular signals to induce proliferation and differentiation [40]. When JNK is activated it can phosphorylate and inactivate the anti-apoptotic protein Bcl-2. Previous data showed that apoptosis induced by 2ME2 is largely dependent on JNK activation [40]. Our data revealed that JNK inhibition largely abrogates the effects of EMBS since cells were no longer blocked in the G_2_/M phase. However, there was still a significant fraction of cells that underwent endoreduplication after 48 h indicating that the JNK pathway may not be the only pathway involved in this process. In addition, the JNK inhibitor had no effect on ROS generation induced by EMBS indicating that the ROS production is an upstream event from the JNK-dependent apoptosis induced by EMBS.

## Conclusion

In summary, we have shown that EMBS increased ROS production in breast cell lines. Furthermore, temporal studies showed that ROS production changed over time after EMBS exposure with initial increases followed by a short, but statistically significant drop in ROS production after which production rates consistently and dramatically increased. This second increase was correlated with mitochondrial membrane depolarisation, G_2_/M block, endoreduplication and increased apoptosis induction. Co-treatment with the free radical scavenger, NAC, abolished these effects induced by EMBS suggesting that the increased ROS production by EMBS is essential for its effects on the cell survival. This study contributes to elucidating the novel oxidative stress-dependent signaling mechanism utilised by sulphamoylated 2ME2 derivatives resulting in mitochondrial damage, cycle arrest, endoreduplication and ultimately apoptosis induction.

## Supporting information

S1 File Raw data and statistical analysis in excel. (XLS)

S1 Fig Thymidine does not influence hydrogen peroxide generation. Hydrogen peroxide was quantified of EMBS-treated cells in the presence or absence of 2 mM thymidine. The graph represents the average fold change between EMBS-treated- and vehicle-treated cells (3 independent experiments with error bars representing s.e.m).
(TIF)

S2 Fig EMBS induces a biphasic hydrogen peroxide response. MDA-MB-231 cells were exposed to 0.4 μM EMBS at the indicated timepoints. Hydrogen peroxide was measured in the presence or absence of NAC. Histograms are representatives of 3 repeats.
(TIF)

S3 Fig EMBS induces a biphasic hydrogen peroxide response. MDA-MB-231 cells were exposed to 0.4 μM EMBS at the indicated timepoints. Superoxide was measured in the presence or absence of NAC. Histograms are representatives of 3 repeats.
(TIF)

S4 Fig EMBS induces mitochondrial membrane depolarisation. MDA-MB-231 cells were exposed to 0.4 μM EMBS at the indicated timepoints. Mitochondrial membrane potential of EMBS-treated cells were analysed using Mitotracker in the presence or absence of 20 mM NAC. Histograms are representatives of 3 repeats.
(TIF)

S5 Fig EMBS induces cell cycle abnormalities, endoreduplication and apoptosis. Cell cycle progression was analysed using PI in cells treated with EMBS alone, EMBS together with NAC or EMBS together with the JNK inhibitor, SP600125. Histograms are representatives of 3 repeats.
(TIF)

S6 Fig EMBS induces apoptosis. MDA-MB-231 cells were exposed to 0.4 μM EMBS at the indicated timepoints. Representative repeat of apoptosis induction demonstrated using Annexin V-FITC and propidium iodide.
(TIF)

S7 Fig Lactate dehydrogenase release: Lactate dehydrogenase levels MCF-7-, MDA-MB-231- and MCF-12A cells exposed to 0.4 μM EMBS-treated for 24 h were compared to vehicle-treated cells. Controls included medium only as background, cells propagated in medium as the low control and cells propagated in medium containing cell lysis solution as the high control. An * demonstrates a statistically significant *P* value <0.05 when compared to vehicle-treated cells.
(TIF)
