# Supplementary figures and images for: A bis-sulphamoylated estradiol derivative induces ROS-dependent cell cycle abnormalities and subsequent apoptosis
Source: PLoS One. 2017 Apr 14;12(4):e0176006. doi: 10.1371/journal.pone.0176006 (PMC5391954; doi:10.1371/journal.pone.0176006)

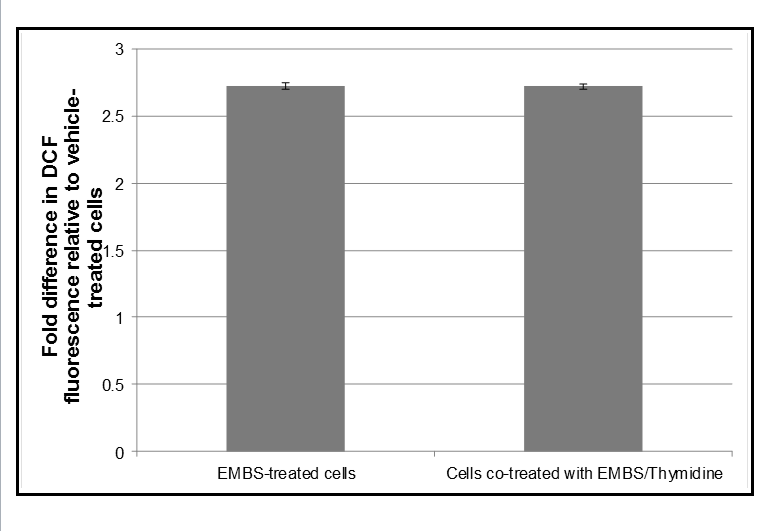

Supplement: S1 Fig — Hydrogen peroxide was quantified of EMBS-treated cells in the presence or absence of 2 mM thymidine. The graph represents the average fold change between EMBS-treated- and vehicle-treated cells (3 independent experiments with error bars representing s.e.m). (TIF) [file pone.0176006.s002.tif]

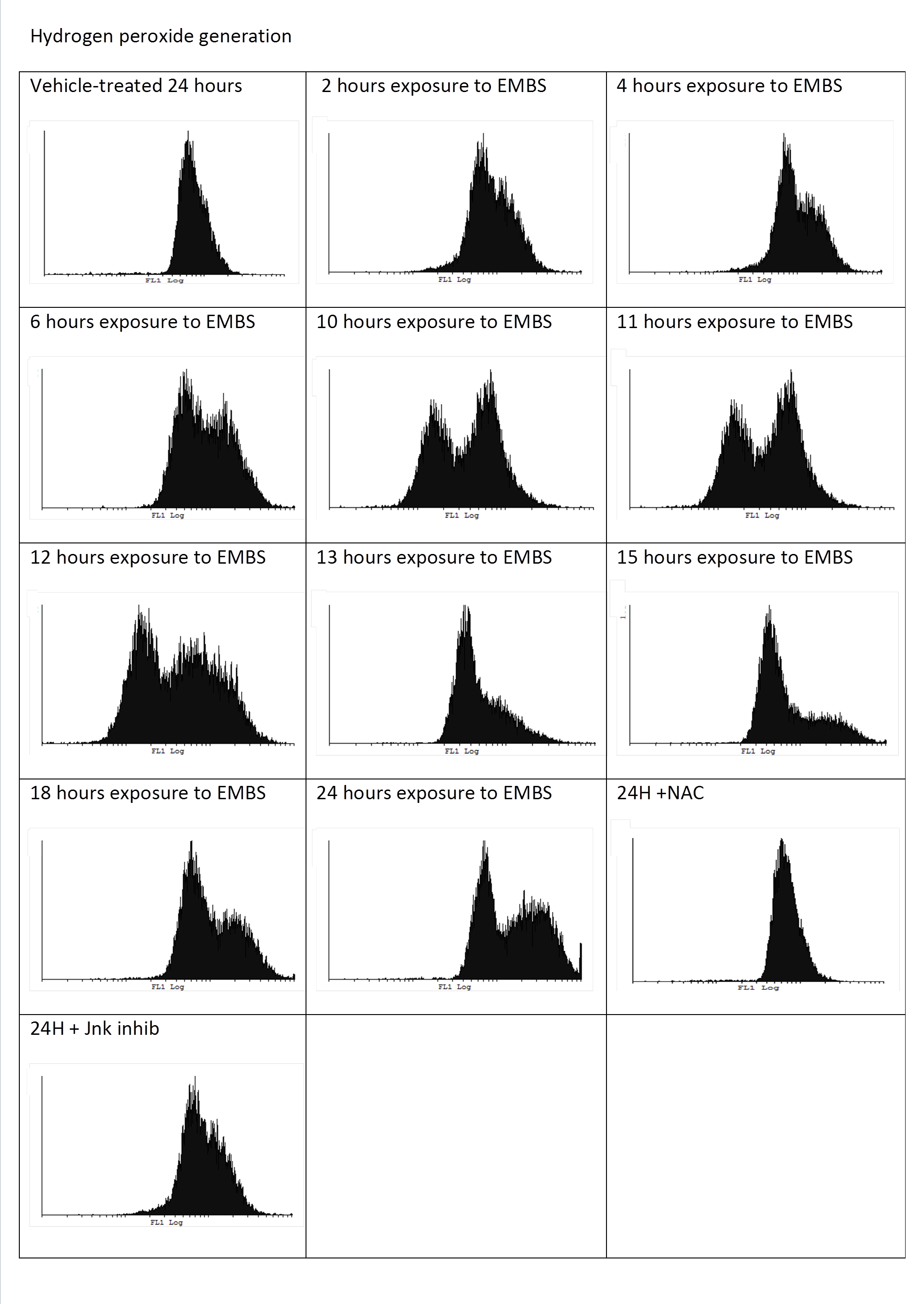

Supplement: S2 Fig — MDA-MB-231 cells were exposed to 0.4 μM EMBS at the indicated timepoints. Hydrogen peroxide was measured in the presence or absence of NAC. Histograms are representatives of 3 repeats. (TIF) [file pone.0176006.s003.tif]

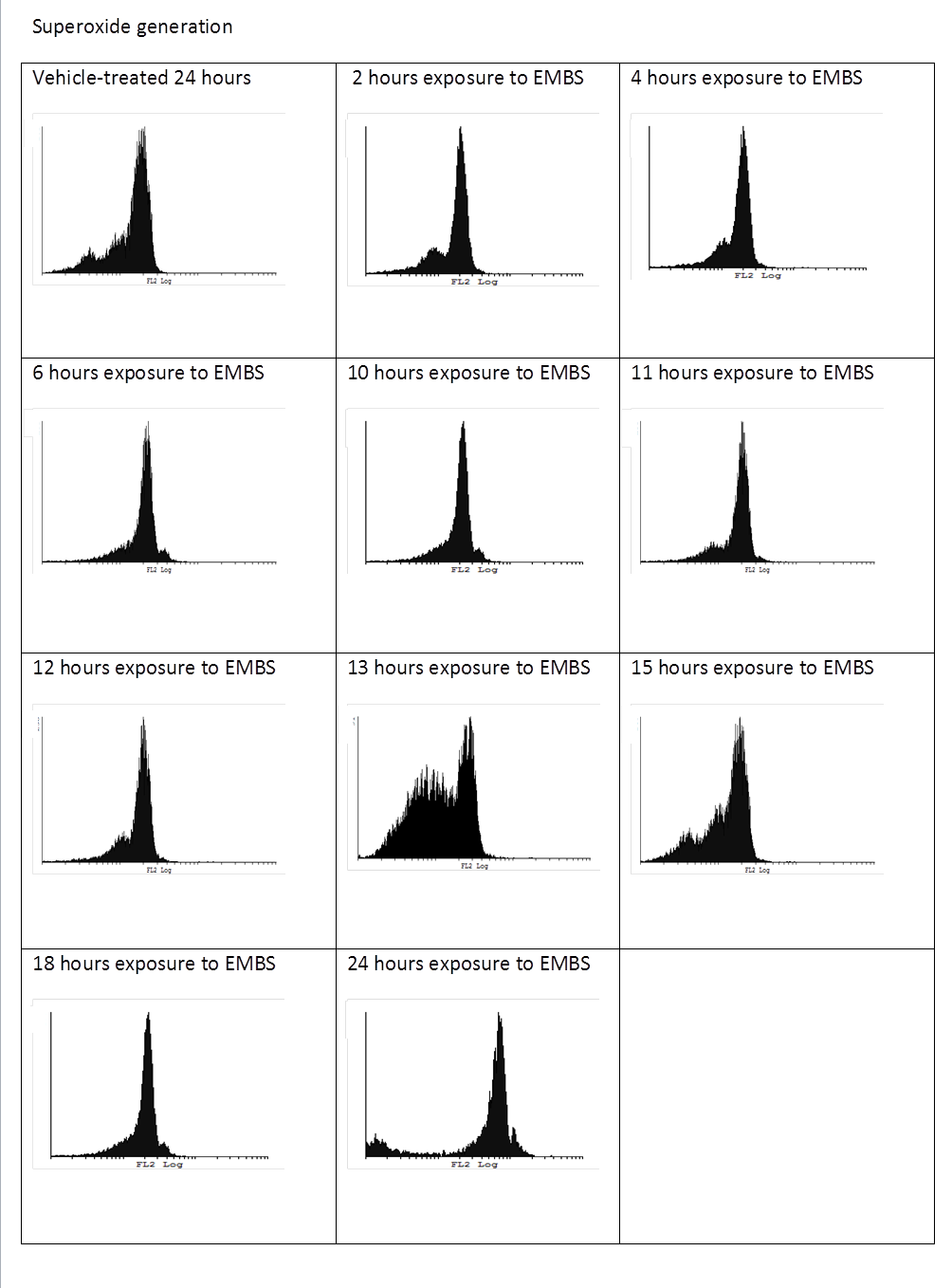

Supplement: S3 Fig — MDA-MB-231 cells were exposed to 0.4 μM EMBS at the indicated timepoints. Superoxide was measured in the presence or absence of NAC. Histograms are representatives of 3 repeats. (TIF) [file pone.0176006.s004.tif]

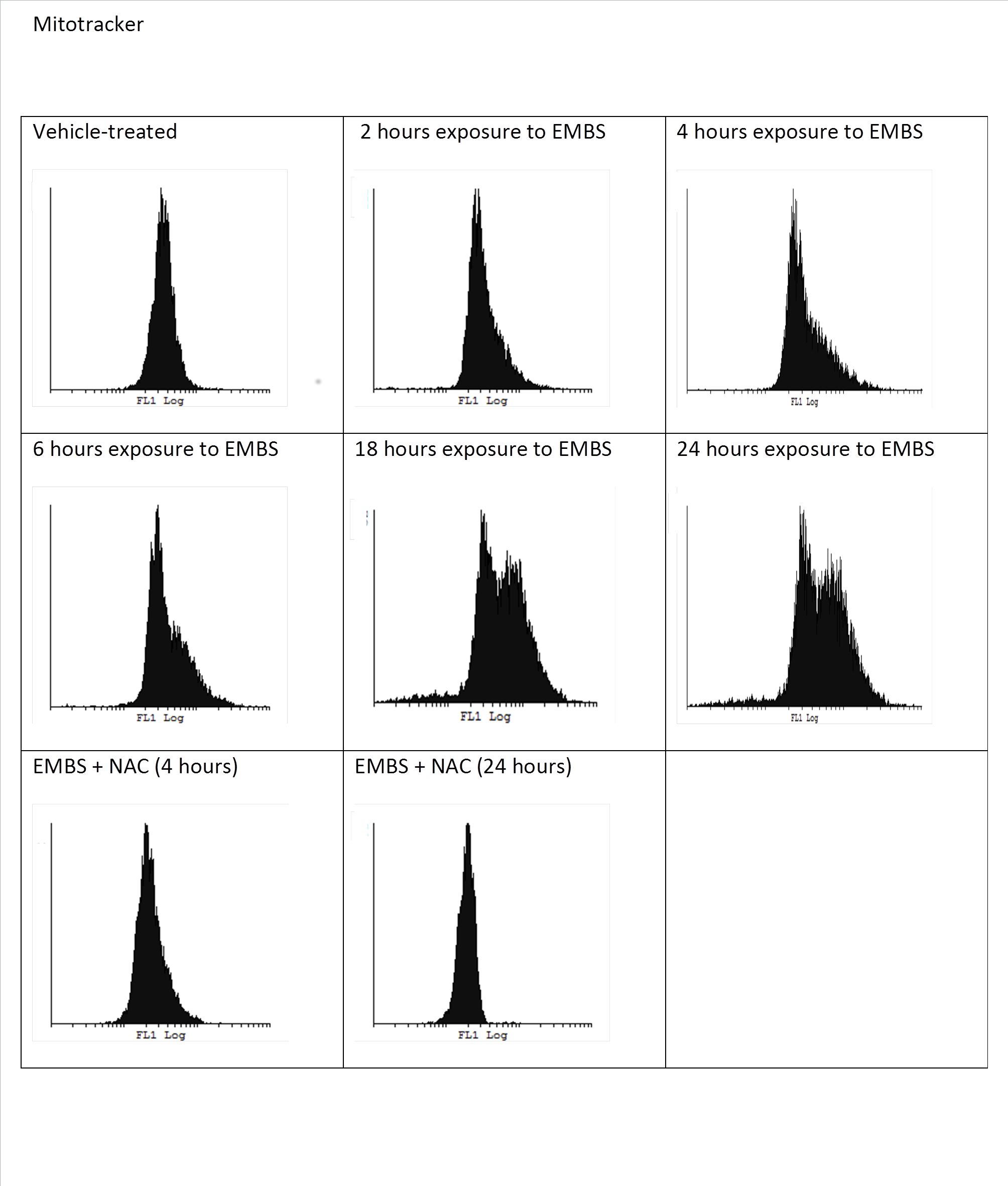

Supplement: S4 Fig — MDA-MB-231 cells were exposed to 0.4 μM EMBS at the indicated timepoints. Mitochondrial membrane potential of EMBS-treated cells were analysed using Mitotracker in the presence or absence of 20 mM NAC. Histograms are representatives of 3 repeats. (TIF) [file pone.0176006.s005.tif]

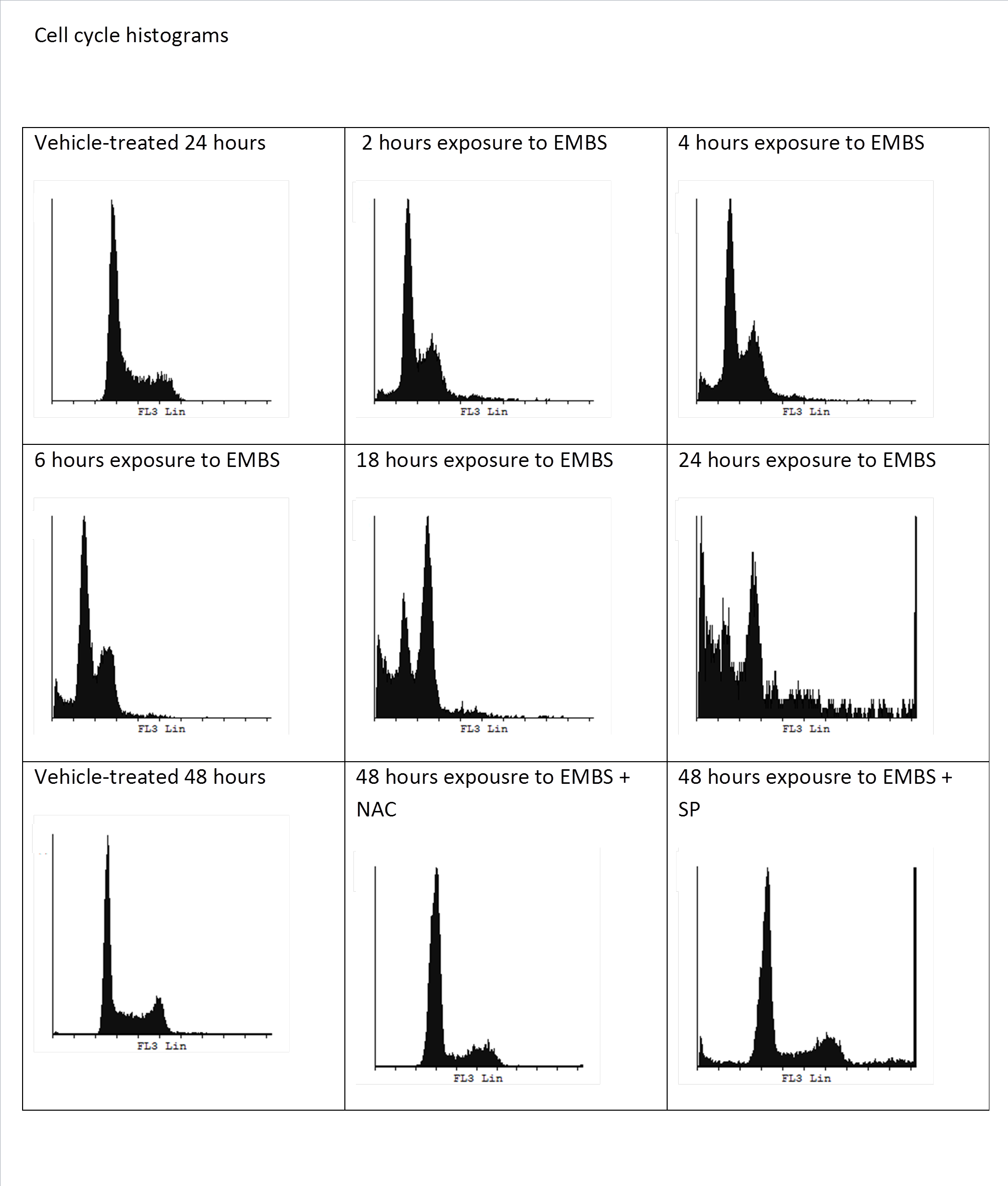

Supplement: S5 Fig — Cell cycle progression was analysed using PI in cells treated with EMBS alone, EMBS together with NAC or EMBS together with the JNK inhibitor, SP600125. Histograms are representatives of 3 repeats. (TIF) [file pone.0176006.s006.tif]

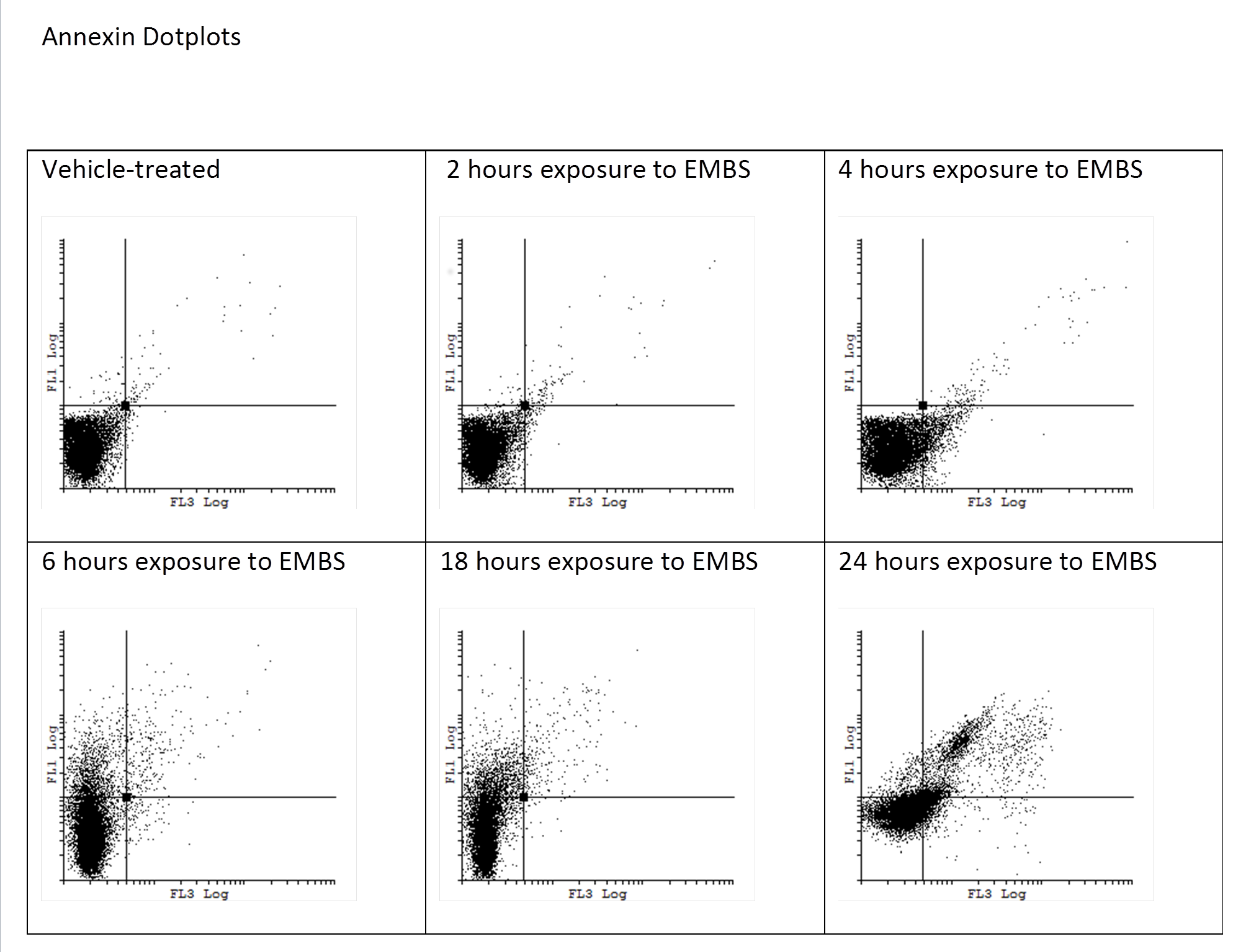

Supplement: S6 Fig — MDA-MB-231 cells were exposed to 0.4 μM EMBS at the indicated timepoints. Representative repeat of apoptosis induction demonstrated using Annexin V-FITC and propidium iodide. (TIF) [file pone.0176006.s007.tif]

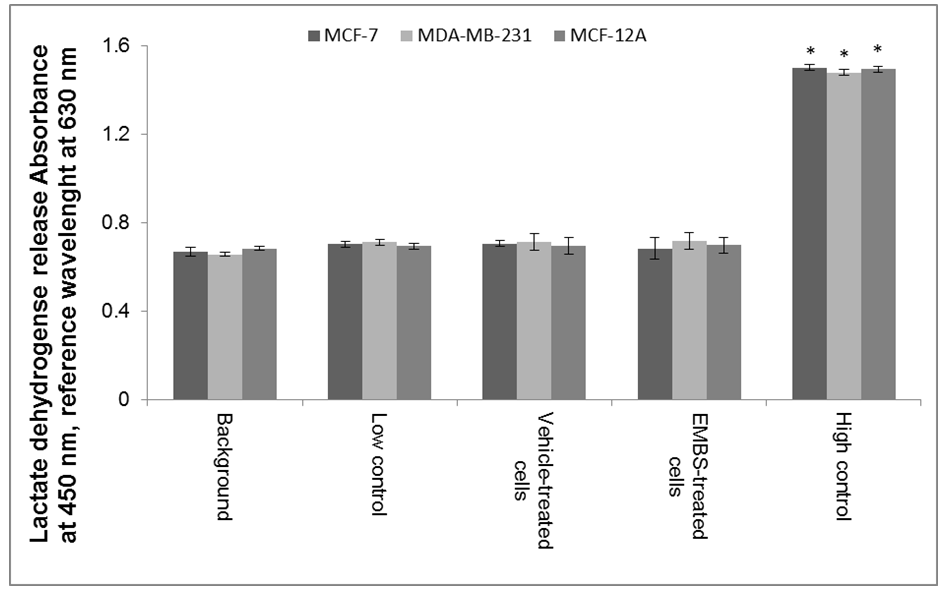

Supplement: S7 Fig — Lactate dehydrogenase levels MCF-7-, MDA-MB-231- and MCF-12A cells exposed to 0.4 μM EMBS-treated for 24 h were compared to vehicle-treated cells. Controls included medium only as background, cells propagated in medium as the low control and cells propagated in medium containing cell lysis solution as the high control. An * demonstrates a statistically significant P value <0.05 when compared to vehicle-treated cells. (TIF) [file pone.0176006.s008.tif]
